# Supplementary material for: Understanding prescribing errors for system optimisation: the technology-related error mechanism classification
Source: BMJ Health Care Inform. 2024 Nov 2;31(1):e100974. doi: 10.1136/bmjhci-2023-100974 (PMC11535662; doi:10.1136/bmjhci-2023-100974)
Supplement: online supplemental file 1 [file bmjhci-31-1-s001.pdf]

SUPPLEMENTARY FILE 1: Guide to applying the Technology-Related Error Mechanism (TREM) classification

Resources required to apply the TREM classification

To determine technology-related prescribing errors and apply the Technology-Related Error Mechanism (TREM) classification, investigators should have access to the following:

- Details of prescribing errors, including the error category (e.g. wrong dose)
- A rating of potential and/or actual harm for each error is beneficial, but not required to assess whether errors are technology-related and the underlying mechanisms of TREs
- Access to the computerised provider order entry (CPOE) records to view the orders with errors
- Access to a testing environment of the CPOE system

Steps to be taken to apply the TREM classification

The steps taken to determine whether an error is technology-related and assign an underlying mechanism are outlined in Table S1, along with examples of investigational questions.

Table S1: Two-step process and examples of investigational questions for applying the Technology-Related Error Mechanism (TREM) classification

Step	Examples of investigational questions to ascertain whether an error was technology-related and the underlying mechanism
1. Review of error in the CPOE	• Was the order based on a prebuilt order sentence/template or order set?<br>• How was the final dose calculated?<br>• Which weight was used in the dose calculator?<br>• Were the medication orders before or after the order being investigated placed by the same prescriber?<br>• Were there discharge prescriptions generated on the day of the error?<br>• Was medication reconciliation conducted on the day of the error?<br>• Was there documentation of the prescriber's intention?
2. Simulate the error in the test environment	• Is there an error in the order sentence or template?<br>• What are the drop-down menu options (e.g. order sentences) in close proximity to the order component selected?<br>• What is the default in an order when no selection is made or when the prescriber hits the 'enter' key?<br>• Is there automation during the ordering process that may have contributed to the error?<br>• What actions were taken by the prescriber to generate the order?

The following pages provide worked examples of error investigations to guide users in the application of the TREM classification:

- Underlying mechanism example 1: System contains an incorrect order sentence or other incorrect configuration
- Underlying mechanism example 2: Construction error
- Underlying mechanism example 3: Editing errors relating to the dose calculator
- Underlying mechanism example 4: Selection error
- Example 5: Error not classified as a TRE

Underlying mechanism example 1: System contains an incorrect order sentence or other incorrect configuration

Clinical error type: Wrong dose

Details: *fentanyl 72 microg, intranasal-both, ONCE*, indicating one spray of 72 microg per nostril giving a total dose of 144 microg. However, this should be one spray of 72 microg in ONE nostril.

**fentanyl (fentanyl nasal spray)**<br>72 microg, Intranasal-Both, ONCE, Stop Date: 03-Jul-16 14:20:00, STAT, Moderate-Severe Pain, Use a 600 microg/2mL intranasal vial to make up the fentanyl nasal spray as per CHW ED guideline.	72 microg @1429

STEP 1: REVIEW ORDER IN THE CPOE

Investigational question 1: Was the order based on a prebuilt order sentence?

On investigation it was noted that the order was based on a prebuilt order sentence, as indicated by the inclusion of information such as the 'Drug Dosage Guidelines' and 'Dose Limits' embedded in the order sentence. These components were usually not completed by a prescriber when constructing an order without an order sentence.

**Details**	
Strength Dose	72
Strength Dose Unit	microg
Target dose	1.5 microg/kg
Actual dose	1.5 microg/kg
Route of Administration	Intranasal-Both
Drug Form	Spray
Frequency	ONCE
First Dose URGENCY	STAT
FIRST DOSE Date/Time	03/07/2016 14:20 AEST
Stop Date/Time	03/07/2016 14:20 AEST
Indication	Moderate-Severe Pain
Special Instructions	Use a 600 microg/2mL intranasal vial to make up the fentanyl nasal spray as per CHW ED guideline.
Drug Dosage Guidelines	CHW ED Intranasal Fentanyl
Dose Limit	100 micrograms

STEP 2: SIMULATE THE ERROR IN THE TEST ENVIRONMENT

Investigational question: Is there an error in the order sentence or template?

On examination of the test environment and the available order sentences it was apparent that the order sentence had been prebuilt with the route 'intranasal-both'.

CONCLUSIONS

The underlying mechanism of the error was '1.2 System contains incorrect order sentence or other incorrect configuration'.

Underlying mechanism example 2: Construction error

Clinical error type: Wrong dose

Details: *Oxycodone 0.6 gm, Oral/Nasogastric, 6 hourly* prescribed resulting in a 1000 times overdose as the units should have been 'mg' (and not 'gm').

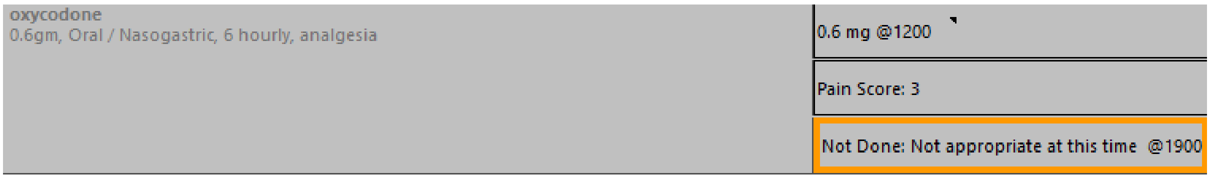

STEP 1: REVIEW ORDER IN THE CPOE

Investigational question 1: Was the order based on a prebuilt order sentence?

On investigation it was noted that the order was not based on a prebuilt order sentence due to the absence of the usual order sentence information such as 'Drug Dosage Guidelines' and 'Dose Limits'. Therefore, the prescriber created the order using the 'free-text' prescribing option, eliminating mechanism categories related to order sentences e.g. Selection errors.

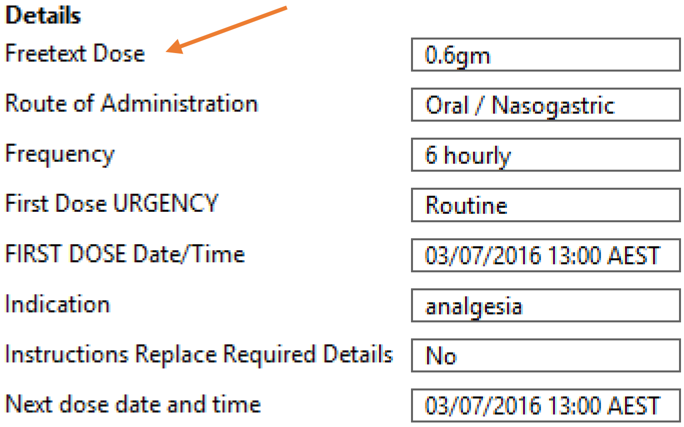

Investigational question 2: Was there information in the order as to how the dose had been determined?

In this case the prescriber did not use the dose calculator to calculate the final dose, as there was no 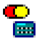 symbol next to the dose and the dose is labelled 'Freetext Dose'. Furthermore, the dose calculator is not presented to the prescriber when creating free text orders.

CONCLUSIONS

The underlying mechanism of the error was '4. Construction errors'.

Underlying mechanism example 3: Editing errors that occur when using the dose calculator

Clinical error type: Wrong dose

Details: *metronidazole IV intermittent 810 mg 12 hourly* prescribed for a 14-year old (weight: 64.6 kg, height: 174 cm). The dose limit for IV metronidazole is 500 mg per dose according to guidelines.

metronidazole
810 mg, IV Intermittent Infusion, 12 hourly, Order Duration: 24 hr(s), Wound infection, Surgical Prophylaxis, Stop Date: 27-Aug-20 15:59:00

STEP 1: REVIEW ORDER IN THE CPOE

Investigational question 1: Was the order based on a prebuilt order sentence?

On investigation it was noted that the order was based on a prebuilt order sentence, as indicated by the inclusion of information such as the 'Drug Dosage Guidelines' and 'Dose Limits' embedded in the order sentence. These components were usually not completed by a prescriber when constructing an order without an order sentence.

Strength Dose	810
Strength Dose Unit	mg
Route of Administration	IV Intermittent Infusion
Frequency	12 hourly
First Dose URGENCY	Routine
FIRST DOSE Date/Time	26/08/2020 16:00 AEST
PRN	No
Duration	24
Duration Unit	hr(s)
Stop Date/Time	27/08/2020 15:59 AEST
AMS Indication	Wound infection
Indication	Surgical Prophylaxis
AMS Approval	intraoperative
Use Patient's Own Medication	No
Order for Future Visit	No
Drug Dosage Guidelines	M4K: 12.5mg/kg/dose q12h
Dose Limit	500mg per Dose

In addition it was noted that the order sentence was part of a PowerPlan (as indicated by the following symbol attached to the order) , and by the pathway description in the 'Additional Info' tab attached to the order:

Comments | History | Validation | Details | Ingredients | **Additional Info** | Results

Ordered As	metronidazole
Start Date/Time	26/08/2020 16:00 AEST
Stop Date/Time	26/08/2020 15:09 AEST
Frequency	12 hourly
Order ID	695983851
**Pathways**	
Post-operative Inpatient Orders [redacted]	231204629

Investigational question 2: Was there information in the order as to how the dose had been determined?

In this case the prescriber had used the dose calculator to calculate the final dose (as indicated by the symbol next to the dose). The dose calculator provided information on the target dose, the weight used to calculate the dose (in this case actual body weight) as well as the pre-programmed standard dose limit of 500 mg.

metronidazole

Dose Values

1) Target dose:	12.5	mg/kg		
2) Calculated dose:	807.5	mg		
3) Dose Adjustment:	807.5	mg	100	%
4) Final dose:	810	mg	12.5387	mg/kg
5) Standard dose:	500	mg	7.7399	mg/kg
6) Rounding rule:	Nearest ten			
7) Adjust Reason:				
8) Route:	IV Intermittent Infusion			

Order Comments

Reference Data

Date of birth:	04/03/2006	(14 Years)		
Sex:	Male			
Race	Neither			
Height:	174.9	cm	Source:	26/08/2020 11:23 174.90 cm Height
Actual weight:	64.6	kg	Source:	26/08/2020 15:07 64.600 kg Weight Dosing
Adjusted weight:	64.6	kg	Adjustment:	Actual (no adjustment)
Serum creatinine:	0	umol/L	Source:	Manually entered
CrCl (est.):			Algorithm:	Schwartz - full term — Missing data
			Weight Used for CrCl:	
Body surface area:	1.77	m2	Algorithm:	Mosteller

STEP 2: SIMULATE THE ERROR IN THE TEST ENVIRONMENT

Investigational question: Is there an error in the order sentence or template?

In this scenario, there was no error in the build or programming of the order sentence. When prescribing using the same order sentence selected by the prescriber and entering the child's weight and height, there was also no error in the dose calculator build.

Investigational question: What are the drop-down menu options (e.g. order sentences) in close proximity to the order component selected?

On examination of the test environment, it was noted that there were two order sentences for metronidazole IV to choose from depending on the child's weight (i.e. for a child weighing less than 40 kg and for a child greater than or equal to 40 kg). However, it was also noted that the order sentences are filtered based on the weight of the child recorded in the record, and thus only one order sentences would have been available to the prescriber, removing the likelihood of a selection error.

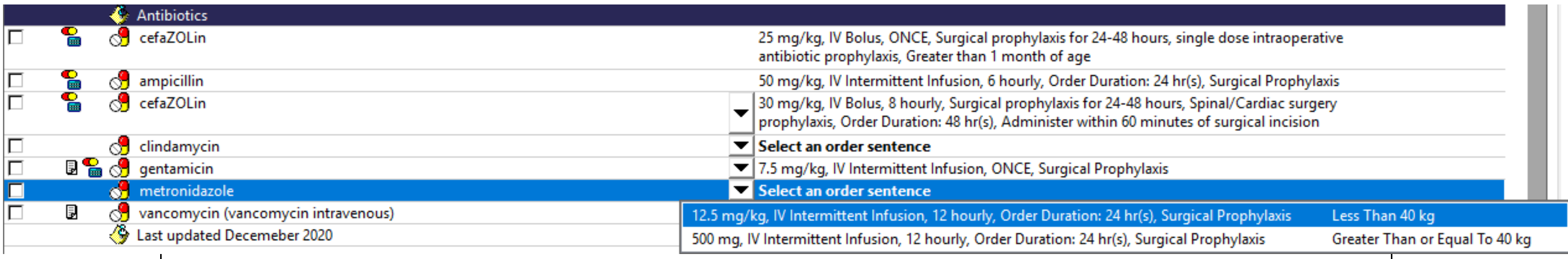

Investigational question: What actions by the prescriber to generate the final dose in the order?

It was determined that the dosage calculator was programmed with the standard dose limit of 500 mg (the 'standard dose'). However, the prescriber had to accept the dose limit by clicking the 'Apply Standard Dose' button. In this scenario, the prescriber instead clicked the 'Apply Dose' button, resulting in the final dose of 810 mg being applied to the order, instead of the intended 500 mg dose.

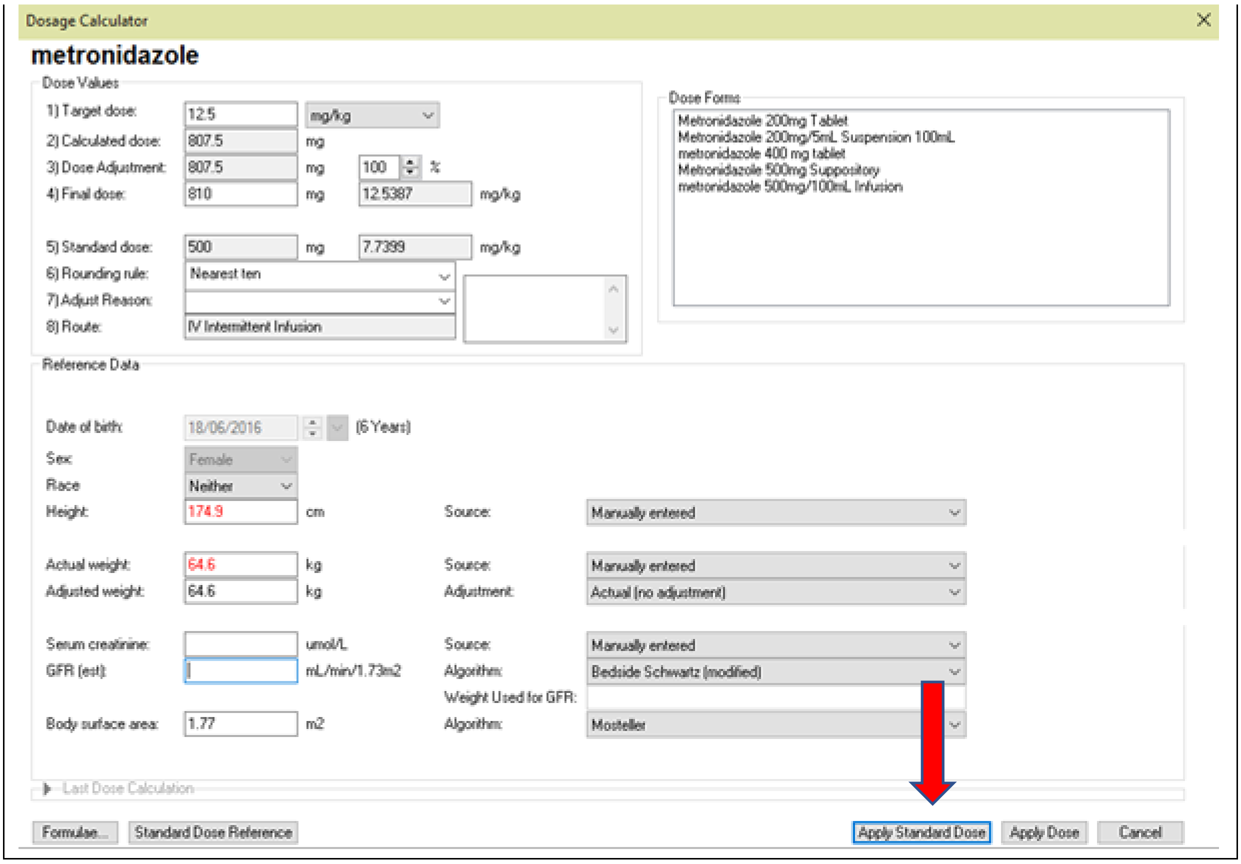

CONCLUSIONS

The underlying mechanism of the error was '5.2 Editing errors that occur when using the dose calculator'.

Example 4: Selection error

Clinical error type: Wrong drug other

Details: *gentamicin inhalation 80 mg BD twice daily indication: cystic fibrosis*. The patient was being treated with intravenous therapy for a urinary tract infection, and did not have cystic fibrosis or require nebulised gentamicin.

gentamicin
80 mg, Nebulised Inhalation, BD, Cystic fibrosis

STEP 1: REVIEW ORDER IN THE CPOE

Investigational question 1: Was the order based on a prebuilt order sentence?

On investigation it was noted that the order was based on a prebuilt order sentence, as indicated by the inclusion of information such as the 'Drug Dosage Guidelines' embedded in the order sentence. These components were usually not completed by a prescriber when constructing an order without an order sentence.

Details

Strength Dose	80
Strength Dose Unit	mg
Route of Administration	Nebulised Inhalation
Drug Form	Ampoule
Frequency	BD
First Dose URGENCY	Routine
FIRST DOSE Date/Time	09/06/2016 20:00 AEST
Indication	Cystic fibrosis
Drug Dosage Guidelines	CHW: 80mg/dose nebulised q12h
Next dose date and time	09/06/2016 20:00 AEST

Investigational question 1: What other medications were prescribed before and after the medication in error?

On investigation it was noted that a second IV medication for urinary tract infection was prescribed at the same time as the order in error. In addition, the incorrect order had been ceased and reordered correctly as 80 mg IV intermittent infusion.

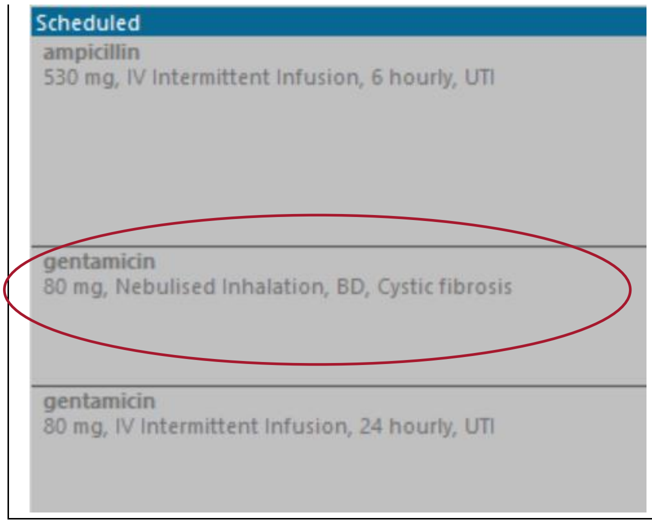

STEP 2: SIMULATE THE ERROR IN THE TEST ENVIRONMENT

Investigational question: What are the drop-down menu options (e.g. order sentences) relating to the order selected?

On investigation it was noted that there were a number of gentamicin options presented to the prescriber to pick from in the drop-down menu. A prescriber intending to prescribe an 80 mg dose may inadvertently see and select the 80 mg nebulised dose rather than a mg/kg order sentence for IV therapy.

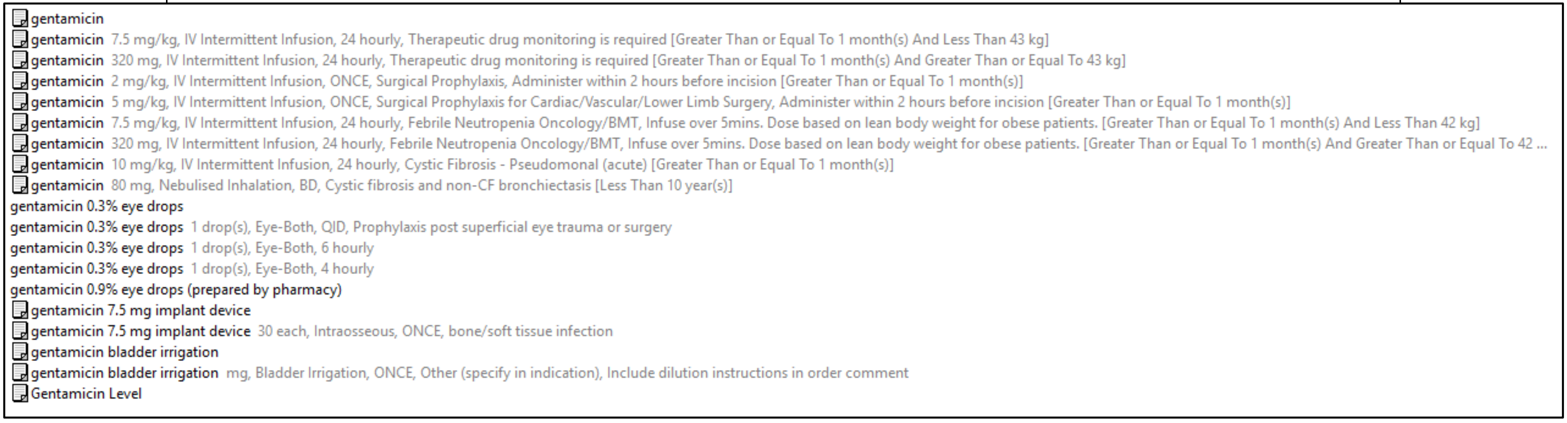

CONCLUSIONS

The underlying mechanism of the error was 3.1 Selection errors when ordering

Example 5: Error not classified as a TRE

Clinical error type: Wrong dose

Details: *paracetamol 440 mg, QID, regular*. Patient is has a weight in the 98.8th percentile for this age, with no height recorded. The weight 50th percentile for this age (5 years old) is 18 kg, giving a recommended dose of 270 mg. 440 mg gives a dose of 24.4 mg/kg.

paracetamol<br>440 mg, Oral, QID, Analgesia, Review after 48 hours.	440 mg @1251
	440 mg @1648

STEP 1: REVIEW ORDER IN THE CPOE

Investigational question 1: Was the order based on a prebuilt order sentence?

On investigation it was noted that the order was based on a prebuilt order sentence, as indicated by the inclusion of information such as the 'Drug Dosage Guidelines' embedded in the order sentence. These components were usually not completed by a prescriber when constructing an order without an order sentence.

Details

Target dose	15 mg/kg
Actual dose	15.17 mg/kg
Strength Dose	440
Strength Dose Unit	mg
Route of Administration	Oral
Frequency	QID
First Dose URGENCY	Routine
FIRST DOSE Date/Time	20/05/2016 12:00 AEST
Indication	Analgesia
Special Instructions	Review after 48 hours.
Dose Limit	90mg/kg/day (up to 4g)
Drug Dosage Guidelines	CHW: 15mg/kg/dose q4-6h
Next dose date and time	20/05/2016 12:00 AEST

Investigational question 2: Was there information in the order as to how the dose had been determined?

It was noted that the dose calculator was used to calculate the dose (as seen by the symbol next to the dose).

On accessing the dose calculator it was noted that no height had been recorded for the child, and the paracetamol dose was based on the child's actual weight, rather than ideal body weight as recommended by the hospital protocol for calculation of paracetamol doses.

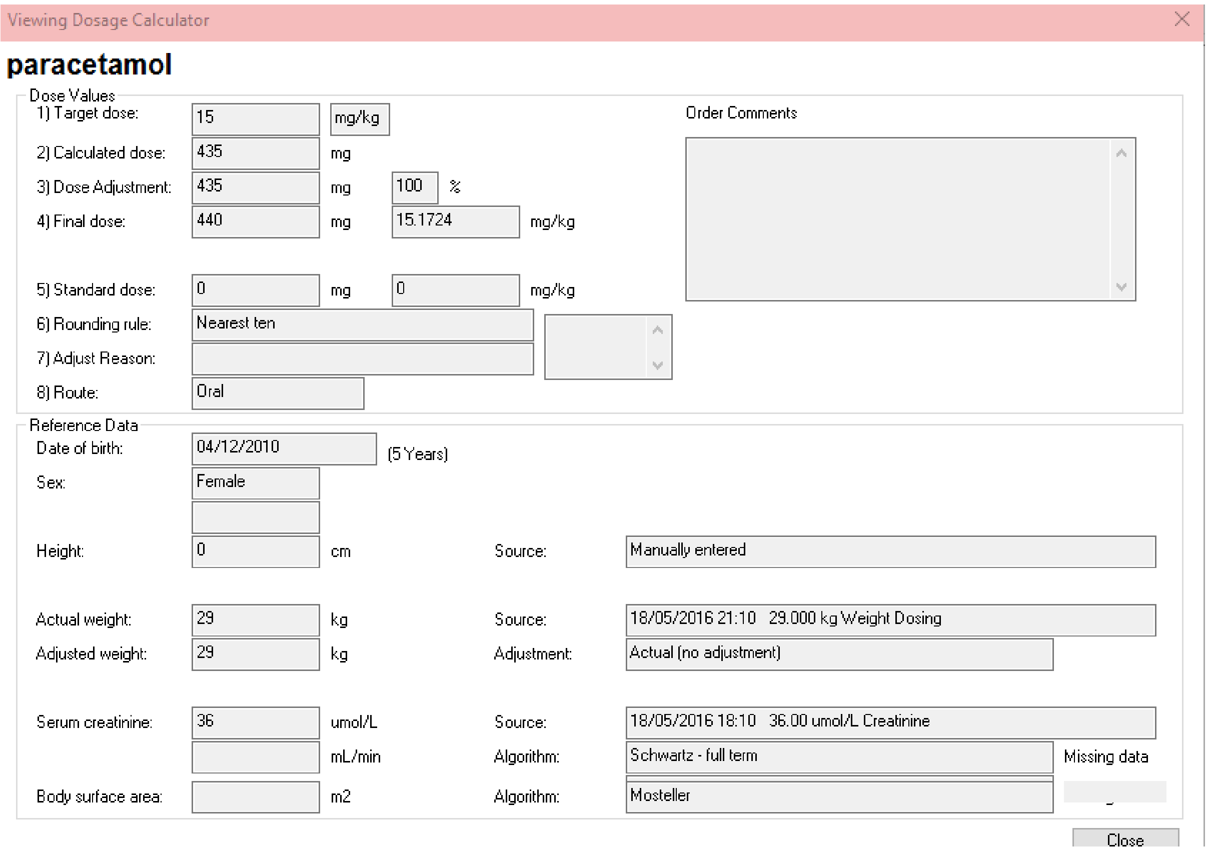

STEP 2: SIMULATE THE ERROR IN THE TEST ENVIRONMENT

Investigational question: Is there an error in the order sentence or template?

In this scenario, there was no error in the build or programming of the order sentence. No further simulation of the error was required.

CONCLUSIONS

It was therefore determined that paracetamol overdose was not caused by the system, rather was an omission on the part of the prescriber in ensuring all the required patient parameters were used for correct calculation of the dose: the error is not technology-related.
